# An Implantable Loop Recorder in the Diagnosis of Cardiac Arrhythmias: The Importance of Drug Treatment in Predicting Pacemaker Requirement

**DOI:** 10.3390/biomedicines14020466

**Published:** 2026-02-19

**Authors:** Jelena Vučković-Filipović, Vladimir Ignjatović, Isidora Stanković, Neda Ćićarić, Vesna Ignjatović, Goran Davidović, Vladimir Miloradović, Violeta Irić-Ćupić, Ivan Simić, Natasa Djordjevic

**Affiliations:** 1 Department of Internal Medicine, Faculty of Medical Sciences, University of Kragujevac, 34000 Kragujevac, Serbia; jelenavufi@gmail.com (J.V.-F.); medicusbg@yahoo.com (G.D.); vanja.miloradovic@gmail.com (V.M.); wwwvikica@ptt.rs (V.I.-Ć.); ivansimickg@gmail.com (I.S.); 2 Clinic for Cardiology, University Clinical Center Kragujevac, 34000 Kragujevac, Serbia; isidora.st.md@gmail.com (I.S.); nedaa.cicaric@gmail.com (N.Ć.); 3 Department of Pharmacology and Toxicology, Faculty of Medical Sciences, University of Kragujevac, 34000 Kragujevac, Serbia; natashadj2002@yahoo.com; 4 Department of Nuclear Medicine, Faculty of Medical Sciences, University of Kragujevac, 34000 Kragujevac, Serbia; vesnaivladaignjatovic@gmail.com

**Keywords:** implantable loop recorder, syncope, cardiac arrhythmia, bradyarrhythmia, pacemaker, oral anticoagulants, diuretics

## Abstract

**Background**: An implantable loop recorder (ILR) represents the gold standard in the diagnosis of cardiac arrhythmias in patients with neurological or cardiac symptoms. Our study aimed to determine the real-world diagnostic effectiveness of ILRs in detecting arrhythmias requiring permanent pacemaker implantation. **Methods**: The study enrolled and followed up for two years 62 ILR recipients from the Cardiology Clinic of the Clinical Center Kragujevac, Serbia. **Results**: The most common indication for pacemaker implantation was pauses in cardiac activity (83%). The use of oral anticoagulants (OR: 11.80; 95% CI: 1.76, 79.4), ACE inhibitors or AT receptor blockers (OR: 3.87; 95% CI: 1.21, 12.35), and diuretics (OR: 5.29; 95% CI: 1.55, 18.04) had a statistically significant impact on the detection of pacemaker-requiring arrhythmias by an ILR. After adjustment for other factors of influence, oral anticoagulants (OR: 7.82; 95% CI: 1.08, 56.9) and diuretics (OR: 3.68; 95% CI: 1.04, 13.00) remained significant in predicting pacemaker requirement in ILR recipients. **Conclusions**: An ILR represents an effective diagnostic approach in detecting cardiac arrhythmias requiring permanent pacemaker implantation, especially in patients treated with oral anticoagulants or diuretics.

## 1. Introduction

Cardiac arrhythmias consist of a diverse group of disorders that vary by origin, prevalence, characteristics, and severity but align in posing a risk of serious complications, such as unexplained syncope [1]. To establish symptom–rhythm correlation, several diagnostic procedures have been proposed, including elective short-term electrocardiogram (ECG) and Holter monitoring, as well as provocative head-up tilt and electrophysiological testing [2,3]. Often being asymptomatic and unpredictable in nature, cardiac arrhythmias have proved to be difficult to diagnose, making elective tests less useful and provocative methods less specific [4,5]. However, even rare arrhythmias can be detected by prolonged monitoring, and coincidence between an abnormal ECG finding and a syncopal episode or palpitations can be considered the gold standard of diagnosis [4].

An implantable loop recorder (ILR) is a small electronic device that can be inserted subcutaneously in the chest to continuously monitor the cardiac rhythm; it will automatically record and store (in the form of an ECG) any significant arrhythmia that occurs during the lifetime of its battery of up to 5 years [6,7,8]. Based on the newest European Stroke Organization guideline, the use of an ILR is recommended for detection of subclinical atrial fibrillation as a possible (and treatable) underlying cause of ischemic stroke or transient ischemic attack of undetermined origin [9]. Similarly, the European Society of Cardiology (ESC) [10] currently recommends using an ILR in the diagnosis of arrhythmias in patients with unexplained syncope, especially in the presence of chronic coronary artery disease and reduced ejection fraction, or cardiomyopathy (class I recommendation). Another established indication for an ILR is unexplained palpitations [11,12], where this method represents a more efficient and more cost-effective diagnostic approach as compared to other conventional strategies [13,14]. Other less common conditions reported to benefit from ILR application include congenital heart disease, cardiac light-chain amyloidosis, obstructive sleep apnea, epilepsy, unexplained falls, and monitoring after coronary artery bypass grafting [15,16].

The efficacy of an ILR in recording concealed cardiac arrhythmias has been demonstrated by several studies. These include a recent systematic review and meta-analysis of randomized controlled trials involving 7237 patients [17], which concluded that ILR-based screening leads to increased detection of incident arrhythmias and increased initiation of appropriate drug therapy. However, the real-world data regarding the diagnostic yield of ILR use in different clinical settings seem to be scarce [8], especially in relation to the conditions requiring treatment involving interventional cardiology procedures [18]. Moreover, while significantly enhancing atrial fibrillation diagnosis in high-risk patients, an ILR does not seem to demonstrate overall usefulness in secondary stroke prevention or mortality reduction [19], indicating the presence of currently unknown modifiers of ILR clinical capacity.

Many patients undergoing ILR implantation receive concomitant pharmacological therapy that can directly influence heart rate, atrioventricular conduction, and arrhythmia expression, potentially affecting the likelihood of arrhythmia detection during prolonged monitoring. Despite the widespread use of such medications in routine clinical practice, the impact of chronic drug therapy on ILR diagnostic yield and subsequent clinical decision-making remains insufficiently explored. Therefore, the aim of our study was to determine the diagnostic effectiveness of an ILR in detecting arrhythmias as an indication for pacemaker or implantable cardioverter–defibrillator (ICD) implantation in patients presenting with transitory neurological or cardiac symptoms, as well as to assess whether concomitant drug therapy and other clinical factors modify the diagnostic yield of an ILR in a real-world setting.

## 2. Materials and Methods

### 2.1. Study Design and Population

This clinical, retrospective, observational study involved patients that received an ILR (Reveal LINQ™, Medtronic, Minneapolis, MN, USA) at the Cardiology Clinic of the Clinical Center Kragujevac from 2019 to 2022. To be included in the study, patients had to fulfill all the inclusion criteria as follows: (1) the presence of neurological or cardiac symptoms as an indication for ILR implantation; (2) completion of a two-year follow-up period or occurrence of a clinical event that required discontinuation of follow-up and further treatment; (3) availability of complete medical documentation. Subjects younger than 18 years of age, as well as pregnant or breastfeeding women, were not considered eligible. All study participants signed an informed consent form prior to entering the study. The study was approved by the relevant Ethics Committee of the Clinical Center Kragujevac, decision No. 01/25-53, dated 20 January 2025.

### 2.2. Follow-Up Protocol

During the follow-up, a standardized diagnostic protocol was applied to all patients. Indications for ILR placement included unexplained syncope or presyncope, recurrent palpitations, suspected bradyarrhythmia, suspected paroxysmal atrial fibrillation, and evaluation following a cryptogenic stroke. Following implantation, ILRs were programmed according to manufacturer recommendations. Device settings typically included automatic detection of pauses, defined as RR intervals longer than 2.5 s; bradycardia, defined as a sustained heart rate ≤40 beats/min (lasting ≥10 s); and atrial fibrillation episodes, defined as an irregular atrial rhythm without discernible P waves lasting ≥2 min. Patients were instructed to activate the device manually in the event of symptoms. ILR data were reviewed at scheduled clinical follow-ups and through remote monitoring alerts. All recorded arrhythmic episodes were evaluated and verified by a cardiologist. For the purpose of clinical decision-making, only rhythm disturbances considered clinically relevant were considered. Pacemaker or ICD implantation was recommended when ILR-confirmed arrhythmias fulfilled guideline-based criteria for permanent device therapy. In patients who subsequently underwent pacemaker or ICD implantation, the ILR device was explanted at the time of permanent device implantation and was not retained thereafter.

### 2.3. Data Collection and Study Outcomes

Demographic and clinical characteristics of the subjects, including gender, age, indication for ILR implantation and associated symptoms, associated cardiovascular and other diseases, chronic therapy, and the result of endocranial computed tomography (CT), were collected from the patients’ medical records. The main study outcome was the presence of arrhythmias detected by the ILR as an indication for implantation of a pacemaker or ICD.

### 2.4. Statistical Analysis

The statistical programs SPSS, version 23, and STATA, version 16, were used for statistical data processing. The normality of the distribution of continuous variables was checked by the Kolmogorov–Smirnov test; in the case of a normal distribution, the mean and standard deviation (SD) were used as measures of central tendency and variability, otherwise the median and interquartile range (IQR) were employed. Categorical variables were presented as absolute numbers and percentages. The influence of independent variables on the outcomes was analyzed by univariable and multivariable binary logistic regression and reported as odds ratios (ORs) with a 95% confidence interval (CI). In cases of unusually large ORs and/or wide 95% CIs, signaling potential imprecision and instability of the estimates, a Firth’s penalized logistic regression model was used. The results are presented in text, tabular or graphical form, with a marginal probability value of less than 0.05.

## 3. Results

A total of 62 patients participated in the study, of whom 24 (38.7%) were women and 38 (61.3%) were men, with the median (IQR) age of 49.5 (35.8–67.8) years. Indications for ILR implantation included syncope (66%), palpitations (27%), stroke (6%), and dizziness (48%), with 27 (44%) patients presenting with at least two of them concurrently. Among those in whom an ILR was implanted for syncope, prodromal symptoms were present in 19 patients, i.e., 46%.

**Figure 1:**
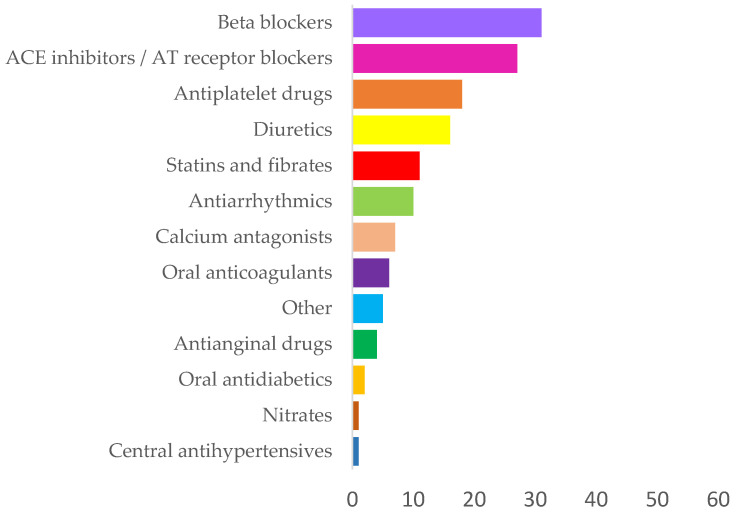
Distribution of drug groups used by the study population.

During the two-year follow-up, syncope occurred occasionally in 43 (69%) patients, while in 21 (49%), these attacks were characterized as frequent. CT of the endocranium was performed in 28 patients, most of whom (93%) had normal findings. Among the respondents, 41 patients (66%) had at least one associated disease (most frequently (61%) arterial hypertension), while 12 (19%) presented with two or more simultaneously. Among the participants, 46 (74%) were on chronic therapy with at least one drug, while 34 (55%) used at least two. The prevalence of drug groups with which the subjects were being treated is shown by Figure 1, while the types of drugs according to individual groups are in Table 1.

The predominance of betablockers and ACE inhibitors/angiotensin receptor blockers reflects the high prevalence of arterial hypertension and cardiovascular comorbidity in the cohort. The use of antiplatelet agents, statins, and antiarrhythmics further indicates a substantial burden of ischemic and rhythm disorders, clinically relevant in patients with recurrent syncope.

**Figure 2:**
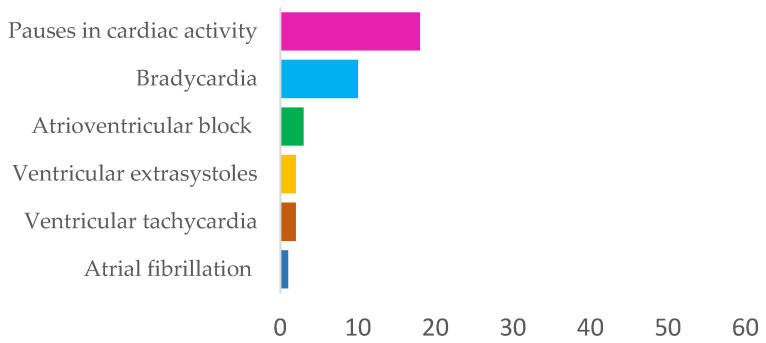
Distribution of cardiac rhythm disorders in the study population.

By the end of the monitoring, coronary artery disease was diagnosed in 10 subjects (16%), hypertrophic myocardiopathy in 6 (10%), and isolated aortic stenosis in 2 (3%) patients. Based on the electrocardiographic recording by the ILR, in 24 patients, which made up 36% of the study population, some form of cardiac rhythm disorder was observed, of which bradyarrhythmias were the most common (Figure 2). Bradycardia (less than 60 heartbeats per min), pauses in cardiac activity (more than 2.5 s between consecutive heartbeats) and atrioventricular block (PR interval longer than 0.2 s or missing QRS complex on ECG) were observed in a total of 20 subjects (32%), of whom 11 (18%) had at least two different arrhythmias detected.

Tachyarrhythmias such as ventricular tachycardia, ventricular extrasystoles, and atrial fibrillation were significantly less common: at least one of them was observed in only 4 patients (6%), while more than two were detected in only one patient.

Bradyarrhythmic events, particularly pauses in cardiac activity and bradycardia, predominated, suggesting a conduction-related mechanism as a major contributor to syncope in this cohort. High-grade atrioventricular block and ventricular arrhythmias were less frequent, while atrial fibrillation was rare, indicating that transient slowing or interruption of cardiac rhythm rather than sustained tachyarrhythmia was the prevailing electrophysiological pattern.

**Figure 3:**
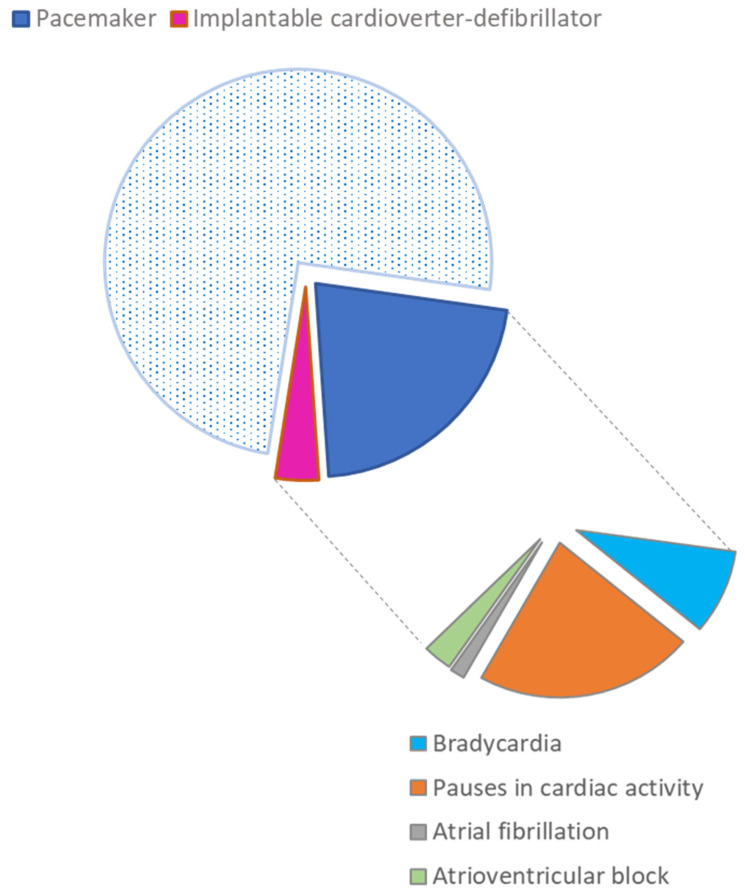
Cardiac rhythm disorders as indications for pacemaker implantation in the study population.

Due to arrhythmia detected by the ILR, in a total of 21 (34%) patients, a pacemaker or ICD was implanted. The distribution of cardiac rhythm disorders as an indication for a pacemaker is shown in Figure 3.

Pacemaker implantation was predominantly indicated for bradyarrhythmic disorders, particularly pauses in cardiac activity and bradycardia, underscoring conduction system dysfunction as the principal therapeutic target in this cohort. Atrioventricular block accounted for a smaller proportion of cases, while atrial fibrillation was rarely an isolated indication, reinforcing that symptomatic bradyarrhythmia rather than tachyarrhythmia drove device therapy.

The most common indication for pacemaker implantation was pauses in cardiac activity (83%), followed by bradycardia (33%); atrioventricular block (11%) and atrial fibrillation (6%) were significantly less common (some of the patients presented with more than one type of arrhythmia). An ICD was inserted in only three patients.

Univariable logistic regression analysis showed that only chronic use of oral anticoagulants, ACE inhibitors or AT receptor blockers, and diuretics had a statistically significant impact on the detection of pacemaker-requiring arrhythmias by the ILR (Table 2).

Since the conventional logistic regression analysis yielded extreme OR estimates, the effect of oral anticoagulants was further assessed with Firth’s penalized logistic regression model. The model showed good overall significance (Wald χ^2^(1) = 6.45; *p* = 0.011) and confirmed the observed significant association of oral anticoagulant use with pacemaker implantation (OR: 11.80; 95% CI: 1.76–79.4).

Multivariable logistic regression with stepwise elimination showed that, when the model was adjusted for the influence of other variables, only the use of oral coagulants and diuretics retained statistical significance (Table 3). In linear regression diagnostics, both predictors showed high tolerance values (0.907) and low variance inflation factor (VIF) values (1.103), indicating no evidence of multicollinearity.

The final model showed statistical significance (χ^2^(2) = 12.647; *p* = 0.002), explained 26.4% of the variance in the outcome, and correctly classified 77.4% of cases. TheHosmer–Lemeshow test (χ^2^ = 0.078; *p* = 0.780) indicated good model calibration, with no significant difference between observed and predicted probabilities. To evaluate the ability to detect the observed associations in the logistic regression model, posthoc power analysis was performed. With a total sample size of 62 per group and α = 0.05, the estimated power to detect the effects of oral anticoagulants and diuretics using a two-sample proportion test was 100% and 84%, respectively.

Due to an extremely wide 95% CI and the relatively low number of events per predictor, the model was retested using Firth’s penalized likelihood regression (Table 4).

The result suggests that patients on oral anticoagulants or diuretics are more likely to be diagnosed by the ILR with arrhythmias as an indication for pacemaker implantation.

## 4. Discussion

In the present study, more than one third of patients with inserted ILRs due to transitory neurological or cardiac symptoms were diagnosed with cardiac arrhythmia and consequently implanted with a pacemaker or an ICD. The patients treated with either oral anticoagulants or diuretics, and possibly also with ACE inhibitors or AT receptor blockers, had significantly higher odds of suffering from pacemaker-requiring arrhythmia. To the best of our knowledge, this is the first study to show that the use of certain drugs affects the detection of pacemaker-requiring arrhythmias by an ILR.

Since 1990, when it was first introduced [20], the ILR has evolved into a small, minimally invasive device with high diagnostic accuracy and low complication rates, making it particularly suitable for prolonged rhythm monitoring in patients with unexplained syncope [21,22]. Over the last couple of decades, the utility of the ILR in the evaluation of unexplained syncope or palpitations has often been compared and almost invariably shown to be superior to other conventional methods [14]. In one of the earliest randomized controlled studies, which included 60 patients with unexplained syncope and employed a partial cross-over design, an ILR-based 1-year-long monitoring strategy was deemed more likely to provide a diagnosis than short-term (up to 4 weeks) ECG monitoring followed by tilt and electrophysiological testing, with underlying arrythmias being detected by the ILR and alternative strategy in 55% and 19% of the patients, respectively [3]. A few years later, in 201 patients suffering from recurrent syncope and randomized to either an ILR or conventional investigation group, 6 months of follow-up revealed that an ECG diagnosis was made in 33% by the former, as opposed to only 4% by the latter approach [5]. In a recent open-label, randomized controlled trial, involving 30 young patients with a structurally normal heart but experiencing recurrent unexplained palpitations or syncope, 1 year of follow-up showed that the implantation of an ILR provides a significantly higher detection rate than conventional extended Holter monitoring (71.5% vs. 18.7%) [14]. These findings are consistent with numerous cohort and registry data resultsconfirming the superior diagnostic yield of an ILR compared with conventional strategies [8,23]. In our study, after 2 years of follow-up, cardiac arrhythmias were diagnosed by the ILR in 36% of the patients. This conforms well to the previous reports and indicates ILR real-world effectiveness. Importantly, this diagnostic yield translated directly into therapeutic consequences, as a substantial proportion of detected arrhythmias resulted in device implantation. In line with many other similar studies [14,18,24], the most frequent cardiac arrhythmias we observed were pauses in cardiac activity and bradycardia.

Previous investigations have shown that the early use of an ILR in patients with unexplained syncope also results in lower healthcare-related costs compared with the conventional evaluation approach [5], especially in populations at higher risk of potentially dangerous, but treatable diseases [25]. Based on the ESC classification, there are five major categories of syncope, of which the cardiac arrhythmia-related type has been associated with the highest one-year overall mortality rate of up to 33% [16]. Interestingly, of all the treatments prescribed to treat syncope, only cardiac pacing has been consistently associated with an improvement of symptoms [5]. As the management and the outcome of up to 90% of all ILR-detected symptomatic bradyarrhythmias largely depends on pacemaker implantation [26,27], in the present study our main goal was to assess the efficacy of the ILR in detecting arrhythmias requiring permanent pacemaker implantation. By focusing on pacemaker-requiring arrhythmias rather than arrhythmia detection alone, our study emphasizes clinically actionable diagnoses, thereby aligning ILR findings with therapeutic decision-making. As expected, the most frequent indications for a pacemaker insertion that we detected were pauses in cardiac activity, followed by bradycardia. Our results comply with the findings of the most recent ILR-related systematic review and meta-analysis, which reported sinus node dysfunction (either sinus pause or sinus bradycardia) as the most common pacemaker-managed arrhythmias [18,28].

Nevertheless, not all ILR recipients with bradyarrhythmia-related syncope seem to benefit equally from a permanent pacemaker. Timely identification of pacemaker implantation predictors, combined with early ILR application, could reduce morbidity and mortality, as well as the cost of the treatment [29]. So far, many factors have been reported to independently predict pacemaker requirement, including older age, obesity, diabetes, and hypertension [18,28,29,30,31]. However, in our study neither demographics (such as gender and age) nor medical conditions (comorbidities and ILR indications) were observed to significantly affect the odds of pacemaker implantation.

Regarding gender, our results are in line with most of the available studies [18], while rare observations of the opposite resultsare usually considered as an incidence finding [29]. However, in terms of age, previous studies are almost completely consistent in classifying elderly patients (older than 65 or 75) as more likely pacemaker recipients [18,32]; as possible explanations they offered age-associated degenerative fibrosis of the cardiac conduction system or other covariables usually associated with aging, such as polypharmacy, electrolyte imbalance, prior cardiac disease, and surgery. In our study, we did observe a tendency toward a positive age–pacemaker correlation, yet we did not find it to be significant. This finding should be interpreted cautiously. The discrepancy between our results and the majority of other reports most likely ensues from the difference in the age structure of the patients (the medium age of our study group was <50 years, as opposed to others dealing with older populations [29,30,31,33]), as well as from the small number of our study subjects belonging to the older age group. Although we are not completely unique in failing to correlate age with pacemaker requirement, it is worth noting that other studies with similar conclusions actually experienced similar limitations [28]. Therefore, we believe that adjusting the age structure of the population, together with increasing the population size, would likely improve the validity of future studies and the accuracy of their findings.

As for the comorbidities and ILR indications, the lack of association between any of them and permanent pacemaker implantation in our study population mostly corresponds to the available literature. Namely, in the recent systematic review and meta-analysis evaluating eight different studies and 1007 ILR recipients [18], arterial hypertension was associated with a higher risk of pacemaker requirement. However, of eight studies evaluated therein, four that covered European populations differed in terms of the predictive value of hypertension: two of them did not find it relevant [29,31], while in the other two, its significance, detected by univariable analysis, was lost after controlling for other factors of influence [28,33]. Similarly, the history of diabetes does not seem to affect the odds of pacemaker requirement either [28,29,30,31], with only one study suggesting the opposite and assuming low tolerance to bradycardia due to diabetic neuropathy to be the reason for their conclusion. Based on the available literature, the role of both arterial hypertension and diabetes in predicting the need for permanent pacemaker implantation thus seems unlikely, but, having in mind the overall prevalence of these diseases, further investigations are welcome. Other comorbidities and ILR indications investigated in our study were either not explored before [18] or did not prove to be important for the prediction of pacemaker requirement in ILR recipients [28,30,31,33].

Conversely, we observed a strong association between the use of certain drugs and pacemaker insertion in patients diagnosed with cardiac arrhythmias. Namely, three out of four patients in our study were treated with at least one drug, with every tenth, every fourth, and almost every second patient being on therapy with oral anticoagulants, diuretics, and ACE inhibitors or AT receptor blockers, respectively. Our results showed that the treatment of ILR recipients with any of these three drug groups is associated with higher odds of having a pacemaker implanted. While these associations are directly supported by our data, the mechanisms discussed below should be regarded as hypothetical. Our conclusions proved to be difficult to put into the context of other investigations, as there are not many previous studies dealing with the predictive value of concomitant medications in this regard.

In relation to renin–angiotensin system (RAS) inhibitors, their role as potential pacemaker implantation predictors was previously assessed, but no significant association was reported [31]. It is well known that RAS inhibitors have little to no effect on heart rate in healthy subjects [34]. However, when used in congestive heart failure [35], these drugs can improve vagal reactivity and lead to baroreflex-mediated bradycardia [36], which could theoretically contribute to clinically relevant bradyarrhythmias in susceptible patients. Moreover, in specific circumstances such as bilateral renal artery stenosis, ACE inhibitors can cause a decline in renal function and renal failure [37,38], which have been reported as independent predictive factors for pacemaker implantation [28]. Finally, these drugs are known to cause hyperkalemia [38,39], which, if severe, can result in both bradycardia [40] and palpitations [41]. These pathophysiological considerations remain speculative and cannot be directly inferred from our data. It should be mentioned that the association between the use of ACE inhibitors or AT receptor blockers and the higher odds of pacemaker requirement detected in our study lost its significance after controlling for other factors of influence. Our observations are in line with a previous report by Huemer et al. [28], who identified the use of AT blockers as predictive of pacemaker implantation in ILR recipients, but their significance dropped after joint comparison with other more important factors of influence. Our hypotheses are generated from an observational study on a small sample of patients, where confounding could be the most probable explanation. Therefore, additional investigations are warranted to confirm the observed effect.

On the contrary, the influence of oral anticoagulants and diuretics, identified as predictive of pacemaker insertions in ILR recipients, remained significant even after multivariable analysis, becoming the only confirmed pacemaker predictors in our study. Regarding oral anticoagulants, both vitamin K and non-vitamin Kantagonists were found to be regularly used by a number of study participants. There is no clear causal relation between oral anticoagulants and pauses in cardiac activity or bradycardia, as these drugs are not typically known to cause arrhythmias [42]. However, one of the most common sustained cardiac arrhythmias, i.e., atrial fibrillation, represents an indication for treatment with oral anticoagulants, due to their high efficacy in the prevention of impending thromboembolism and the consequent embolic stroke [42]. As atrial fibrillation often exhibits palpitations [43] and commonly occurs in patients with sinus node disfunction [44], it is possible that oral anticoagulant use serves as a clinical marker of underlying rhythm disorders, rather than the disorders being a direct effect of the drugs themselves.

In regard to diuretics, their use in relation to pacemaker implantation requirement was only sporadically investigated until now, with the results mainly indicating the lack of any association. In a retrospective single-center study by Huemer et al. [28] that involved 106 patients with an ILR, diuretics were not identified as predictors of pacemaker implantation. The same conclusion was drawn earlier by Palmisano et al. [31] in a prospective multi-center study investigating 56 ILR recipients, with a follow-up of almost 2 years. However, it is well known that diuretics (except for potassium-sparing diuretics, which were not used by our study subjects) often lead to hypokalemia [45], due to excessive excretion of potassium as a “side effect” of their mechanisms of action [46]. In the heart, potassium depletion causes reduction in repolarization reserve and intracellular Na^+^ and Ca^2+^ overload [46], which can generate a wide variety of arrhythmias, including bradycardia [47]. While we did not measure serum potassium levels, hypokalemia represents a biologically plausible mechanism that may partly explain our findings. Although the studies on patients without an ILR cannot be fully comparable with those with an ILR inserted, it is worth mentioning that the use of diuretics has been associated with pacemaker requirement in the presence of cardiac amyloidosis [48], while lower potassium levels were correlated with higher odds of permanent pacemaker implantation in patients with drug-related atrioventricular block [49], both suggesting the possibility of a similar effect in others diagnosed with conduction system impairment. Still, our study remains the first to detect the association between the use of oral anticoagulants and diuretics and permanent pacemaker implantation in ILR recipients. Overall, our results highlight the role of an ILR as a valuable and effective diagnostic tool in routine clinical practice for identifying clinically relevant arrhythmias that require permanent pacemaker implantation, particularly in patients treated with oral anticoagulants or diuretics. In line with current guideline-directed recommendations that favor prolonged rhythm monitoring when intermittent arrhythmias are suspected but not detected by conventional methods, our study points toward early and selective use of an ILR to facilitate timely and appropriate therapeutic intervention.

It should be noted, however, that this study suffers from several limitations, including small sample size (the study is particularly underpowered for rarer outcomes, such as ICD) that limits the robustness of multivariable analyses and increases the risk of overfitting and unstable estimates, a patient cohort younger than that in typical ILR studies, the lack of a control group subjected to alternative diagnostic methods, the lack of serum potassium level analysis, single-center bias, potential selection bias, the lack of blinding in event adjudication, and the lack of other demographic and medical data that might affect diagnostic efficacy of the ILR and the pacemaker requirements.

## 5. Conclusions

Our study shows that an ILR represents an effective diagnostic approach in detecting cardiac arrhythmias requiring permanent pacemaker implantation, especially in patients treated with oral anticoagulants or diuretics. The relevance of previous treatment with ACE inhibitors or AT receptor blockers remains to be confirmed in the future.

## Figures and Tables

**Table 1:** Types of medications used during follow-up.

Antiplatelet Drugs	Acetylsalicylic Acidand Clopidogrel
Oral anticoagulants	Warfarin, apixaban, rivaroxaban, dabigatran, edoxaban
ACE inhibitors and AT receptor blockers	Enalapril, ramipril, perindopril, lisinopril, valsartan
Beta blockers	Propranolol, metoprolol, bisoprolol, nebivolol, carvedilol, sotalol
Calcium antagonists	Amlodipine
Diuretics	Furosemide, torsemide, indapamide, hydrochlorothiazide
Antiarrhythmics	Amiodarone, flecainide, propafenone
Antianginal drugs	Trimetazidine, ivabradine
Central antihypertensives	Moxonidine
Nitrates	Isosorbide mononitrate
Oral antidiabetics	Metformin, gliclazide, dapagliflozin
Statins and fibrates	Atorvastatin, rosuvastatin, fenofibrate
Other medications	Escitalopram, levothyroxine, lorazepam, nicergoline, pantoprazole

Medications were categorized into therapeutic groups based on their predominant clinical use during follow-up.

**Table 2:** Parameters of the univariable analysis of the influence of factors on the efficacy of the ILR in determining indications for pacemaker implantation.

	B	SE	Wald χ^2^	df	*p*	OR	95% CI
Demographic characteristics							
Gender	0.325	0.587	0.308	1	0.579	1.385	0.439–4.371
Age	0.033	0.018	3.489	1	0.062	1.033	0.998–1.070
Indications for ILR							
Syncope	0.396	0.612	0.418	1	0.518	1.486	0.447–4.935
Prodromes	0.539	0.574	0.881	1	0.348	1.714	0.556–5.283
Palpitations	−0.384	0.656	0.342	1	0.559	0.681	0.188–2.466
Stroke	−0.218	1.190	0.034	1	0.855	0.804	0.078–8.285
Dizziness	0.405	0.563	0.519	1	0.471	1.500	0.498–4.519
Associated diseases							
Arterial hypertension	0.681	0.608	1.255	1	0.263	1.976	0.600–6.505
Diabetes	−0.721	0.838	0.741	1	0.389	0.486	0.094–2.512
Anemia	0.928	1.443	0.414	1	0.520	2.529	0.150–42.791
Epilepsy	22.154	40,192.9	0.000	1	1.000	/	/
Other hereditary diseases	−20.38	23,205.4	0.000	1	0.999	/	/
Chronic therapy							
Antiplatelet drugs	−0.950	0.708	1.802	1	0.180	0.387	0.097–1.549
Oral anticoagulants	2.806	1.140	6.054	1	0.014	16.538	1.770–154.55
ACE inhibitors and AT receptor blockers	1.352	0.593	5.209	1	0.022	3.867	1.210–12.352
Beta blockers	−0.314	0.562	0.312	1	0.576	0.730	0.243–2.199
Calcium antagonists	−0.025	0.888	0.001	1	0.977	0.975	0.171–5.555
Diuretics	1.665	0.626	7.070	1	0.008	5.286	1.549–18.035
Antiarrhythmics	0.593	0.717	0.684	1	0.408	1.810	0.444–7.380
Antianginal drugs	−20,404	20,096.5	0.000	1	0.999	/	/
Central antihypertensives	−20.332	40,192.9	0.000	1	1.000	/	/
Nitrates	−20.332	40,192.9	0.000	1	1.000	/	/
Oral antidiabetics	−20.356	28,420.7	0.000	1	0.999	/	/
Statins and fibrates	0.412	0.701	0.346	1	0.556	1.510	0.382–5.966
Other medications	1.435	0.961	2.229	1	0.135	4.200	0.638–27.630

Statistical analysis of the influence of factors on the efficacy of the ILR in determining indications for implantable cardioverter–defibrillator implantation was not conducted due to the small sample size. Abbreviations: ILR, implantable loop recorder; B, the regression coefficient; SE, standard error; Wald χ^2^, Wald test statistic; df, the degree of freedom; *p*, probability; OR, odds ratio; 95% CI, 95% confidence interval for the estimated OR.

**Table 3:** Parameters of the multivariable analysis of the influence of factors on the detection of pacemaker-requiring arrhythmias by the ILR, using multivariable logistic regression with stepwise elimination.

	B	SE	Wald χ^2^	df	*p*	OR	95% CI
Oral anticoagulants	2.407	1.183	4135	1	0.042	11.096	1.091–1112.8
Diuretics	1.364	0.673	4115	1	0.043	3.913	1.047–14.623
Constant	−1.566	0.393	15,868	1	0.000	0.209	

Abbreviations: B, the regression coefficient; SE, standard error; Wald χ^2^, Wald test statistic; df, the degree of freedom; *p*, probability; OR, odds ratio; 95% CI, 95% confidence interval for the estimated OR.

**Table 4:** Parameters of the multivariable analysis of the influence of factors on the detection of pacemaker-requiring arrhythmias by the ILR, using Firth’s penalized likelihood regression.

	B	SE	*p*	OR	95% CI
Oral anticoagulants	2.057	1.012	0.042	7.82	1.08–56.9
Diuretics	1.304	0.644	0.043	3.68	1.04–13.0
Constant	−1.509	0.381	0.000	0.22	

Wald χ^2^(2) = 8.94; *p* = 0.012. Abbreviations: B, the regression coefficient; SE, standard error; Wald χ^2^, Wald test statistic; *p*, probability; OR, odds ratio; 95% CI, 95% confidence interval for the estimated OR.

## Data Availability

Raw data are available at https://doi.org/10.6084/m9.figshare.30178300.v1.

## Abbreviations

The following abbreviations are used in this manuscript:

CT	Computed tomography
ECG	Electrocardiogram
ESC	European Society of Cardiology
ICD	Implantable cardioverter–defibrillator
ILR	Implantable loop recorder
IQR	Interquartile range
SD	Standard deviation
